# Heart Failure-Related Ascites With Low Serum-Ascites Albumin Gradient: Diagnostic Clues From Triphasic Abdominal Computed Tomography

**DOI:** 10.7759/cureus.21251

**Published:** 2022-01-14

**Authors:** Angkawipa Trongtorsak, Veraprapas Kittipibul, Drashti Antala, Qingqing Meng, Sarinya Puwanant

**Affiliations:** 1 Internal Medicine, AMITA Health Saint Francis Hospital, Evanston, USA; 2 Cardiology, Duke University Medical Center, Durham, USA; 3 Cardiovascular Medicine, Chulalongkorn University, Bangkok, THA

**Keywords:** saag, triphasic abdominal computed tomography, low serum-ascites albumin gradient, ascites, heart failure

## Abstract

Serum-ascites albumin gradient (SAAG) is an initial and useful measure to differentiate causes of ascites. High gradient ascites (SAAG >1.1 g/dL) is one of the important features of heart failure. Low gradient ascites in heart failure is relatively rare and needs additional workups to rule out other serious causes, such as malignancy and infection. We herein report a case of a 42-year-old female with low-SAAG ascites from worsening congestive heart failure, which was confirmed to be portal hypertension-originated by triphasic abdominal computed tomography.

## Introduction

Ascites is an abnormal accumulation of fluid in the peritoneal space. The most common cause of ascites is decompensated liver cirrhosis; however, various conditions can also lead to ascites including congestive heart failure [1]. Venous return is impeded in heart failure, resulting in an expansion of venous volume, higher hydrostatic pressure, and later filtration of fluid into the peritoneal cavity [2]. In the past, two types of ascites, exudate and transudate, were classified by the level of total protein concentration in ascitic fluid. Later, serum-ascites albumin gradient (SAAG) was introduced into clinical practice as a more useful parameter to help determine the etiology of ascites as it reflects oncotic-hydrostatic balance [3,4]. A SAAG of 1.1 g/dl or greater indicates the presence of portal hypertension, which is a feature of heart failure-related ascites [5]. Nonetheless, SAAG can be falsely low in a long-standing HF as presented in this case.

## Case presentation

**Figure 1:**
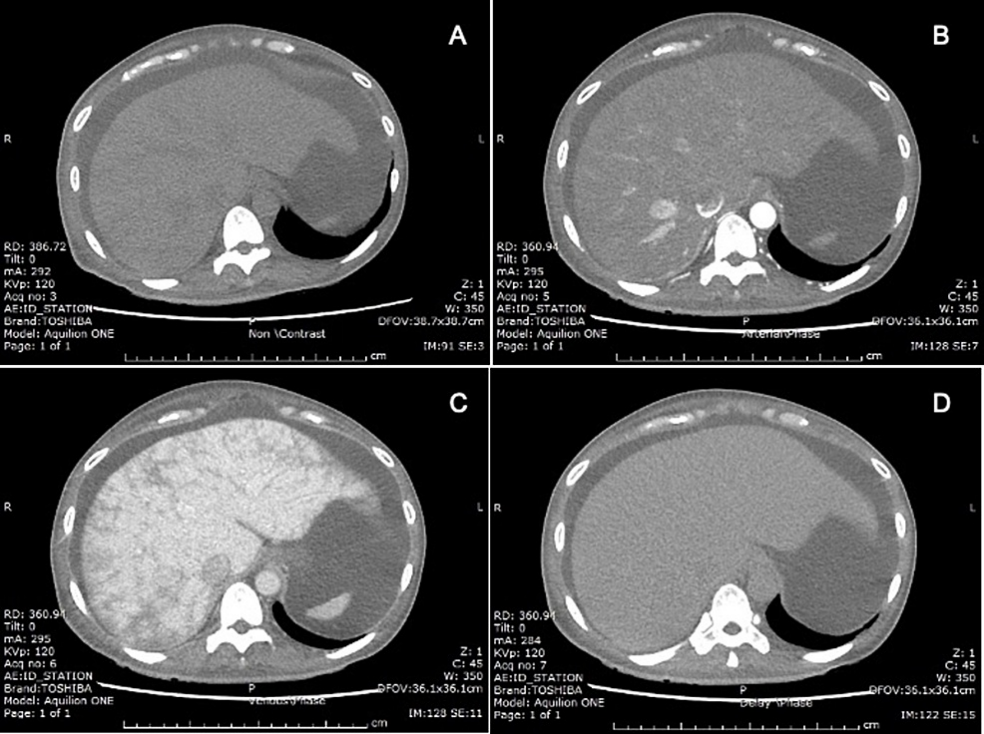
Triphasic abdominal CT scan (A) Non-contrast, (B) arterial phase, (C) portal venous phase, and (D) delayed phase. Hepatomegaly and heterogenous, mottled liver parenchyma with areas of poor enhancement on portal venous phase without demonstrable peritoneal nodule or intra-abdominal lymph node enlargement.

A 42-year-old woman with a history of non-ischemic cardiomyopathy presented to the hospital with progressive dyspnea on exertion and marked abdominal distension over the past four months. On examination, the patient had bilateral lower extremity edema and ascites but no other signs of chronic liver disease. BNP was elevated to 900 pg/mL. Liver enzymes and INR were unremarkable. She was diagnosed with acute decompensated heart failure. Ascites secondary to heart failure was suspected of causing her abdominal distension, for which abdominal paracentesis was done to confirm the etiology. Surprisingly, the ascitic fluid profile showed SAAG of 0.9 g/dL (serum albumin 1.7 g/dL, ascites albumin 0.8 g/dL) and ascites total protein of 4.1 g/dL, which are not typical for ascites from cardiac causes. Ascitic fluid cytology and culture for bacteria and Mycobacterium were all negative. Triphasic abdominal computed tomography (CT) was ordered to exclude other serious conditions such as intra-abdominal cancer and peritoneal disease. The CT scan revealed marked ascites, hepatomegaly, and heterogenous, mottled liver parenchyma with areas of poor enhancement in the portal venous phase without demonstrable peritoneal nodule or intra-abdominal lymph node enlargement (Figure 1). These findings were compatible with passive liver congestion resulting from increased backward flow in congestive heart failure. Transthoracic echocardiography (not shown) substantiated the cardiac cause of the congested liver by demonstrating reduced left ventricular systolic function with an ejection fraction of 16%, an enlarged right atrium and ventricle, impaired right ventricular systolic function, and moderate tricuspid regurgitation. After aggressive diuresis as a treatment for congestive heart failure, ascites improved without additional therapeutic paracentesis.

## Discussion

Heart failure is one of the most common causes of portal hypertension-related ascites, in addition to cirrhosis. Both High SAAG levels (≥1.1 g/dL) and high ascites protein levels (≥2.5 g/dL) are important features of heart failure-related ascites [5]. When the ascitic fluid profile shows a low gradient (SAAG < 1.1 g/dL), portal hypertension-related ascites is less likely. However, SAAG can be falsely low in some conditions as the accuracy ranges around 87.5-97% [3,6-8]. Serum albumin less than 1.1 g/dL, hyperglobulinemia, arterial hypotension/shock, and even long-standing congestive heart failure, as illustrated in the case, may result in unexpectedly low SAAG ascites [9]. The study by Farias et al. showed that 20% (9 out of 44) of heart failure patients were found to have a low SAAG value (<1.1 g/dL). Moreover, they found that those patients had lower serum albumin values compared to those with high gradient ascites. They also suggested that serum BNP may be a useful parameter to rule out heart failure-related ascites with a cutoff of >364 pg/mL (sensitivity 98%, specificity 99%, and diagnostic accuracy 99%) [5].

Further investigation is warranted in patients with low SAAG gradient and unclear etiology. Triphasic abdominal CT can be considered in cases of suspected malignancy or space-occupying lesions in the liver as a cause of ascites. Moreover, triphasic abdominal CT findings of hepatomegaly and a mosaic pattern of liver parenchyma with areas of poor enhancement in the portal venous phase might help establish a diagnosis of heart failure-related ascites in patients with equivocal clinical presentation and laboratory values [10]. Nonetheless, this investigation should not be routinely performed in patients with the typical presentation of ascites due to heart failure.

## Conclusions

One of the important features of heart failure-related ascites is high gradient ascites (SAAG >1.1 g/dL); however, low gradient ascites is not uncommon in patients with long-standing heart failure, especially those with low serum albumin values. For confirmation of heart failure-related ascites, triphasic abdominal CT might be a valuable tool to determine the etiology of ascites in those cases.
